# *Uncitermes almeriae*, a new termite species from Amazonia (Isoptera, Termitidae, Syntermitinae)

**DOI:** 10.3897/zookeys.595.8626

**Published:** 2016-06-02

**Authors:** Tiago F. Carrijo, Joice P. Constantini, Rudolf H. Scheffrahn

**Affiliations:** 1 Museu de Zoologia da Universidade de São Paulo, Cx. Postal 42.494, 04218–970, São Paulo, SP, Brazil; 2 Centro de Ciências Naturais e Humanas, Universidade Federal do ABC, Rua Arcturus, 03, Jardim Antares, 09606-070, São Bernardo do Campo, SP, Brasil; 3 Fort Lauderdale Research and Education Center, Institute for Food and Agricultural Science, 3205 College Avenue Davie, Florida 33314, U.S.A

**Keywords:** Taxonomy, South America, Biological Notes

## Abstract

The Neotropical termite genus *Uncitermes* Rocha & Cancello, 2012 was known from a single species, *Uncitermes teevani* (Emerson, 1925). In this paper a new species, *Uncitermes almeriae* **sp. n.**, is described and illustrated from worker and soldier castes, along with observations on the *Uncitermes* nest. A distribution map with the occurrences of both species is presented. The new species is distinguished from its congener by the presence of short bristles covering the head capsule and frontal tube.

## Introduction

The genus *Uncitermes* Rocha & Cancello, 2012 was described to accommodate the Amazonian species *Uncitermes teevani* (Emerson, 1925), previously included in *Armitermes* Wasmann, 1897. The genus can be distinguished from the other Syntermitinae genera by the strongly recurved soldier mandibles and lack of spines on the margins of pro-, meso- and metanotum, as well as the absence of a projection on the forecoxae (Rocha et al. 2012).

Previously hypothesized as sister group of *Rhynchotermes* Holmgren, 1912 by morphological similarities and a morphological phylogeny (Rocha et al. 2012), molecular data now suggest that *Uncitermes* should be more related to *Armitermes* *sensu stricto*, along with *Embiratermes heterotypus* (Silvestri, 1901) and *Macuxitermes* Cancello & Bandeira, 1992 (Maurício M. Rocha, personal communication).

Herein we describe, from Peruvian and Ecuadorian Amazonia, a second species of the genus, *Uncitermes almeriae* sp. n. The soldier and worker of the new species are described and an updated distribution map is given for both species.

## Material and methods

The institutional collections acronyms cited in this paper are: MZUSP: Museu de Zoologia da Universidade de São Paulo, São Paulo, Brazil; UF: Fort Lauderdale Research and Education Center, University of Florida, Davie, Florida, United States.

Images of the head capsule and digestive tube were taken with a Leica M205C stereomicroscope attached to a Leica DFC 425 digital camera. Specimens were placed in a plastic Petri dish containing 70% ethanol gel (Purell® hand sanitizer). A mirror was placed underneath the dish to highlight pilosity.

Mandibles and enteric valves were mounted on slides with PVA mounting medium (BioQuip #6371A) and the images were taken with a Leica DM5500B compound microscope attached to a Leica DFC 425 camera. All images were composed of multiple photomicrographs taken at different focal planes that were merged with Helicon Focus 6 software. Measurements were taken with an ocular micrometer fitted to an Olympus SZX9 stereomicroscope. Terms used for pilosity are comparative: long bristles are erect hairs with well-marked bases; short bristles are smaller than long bristles; and thick bristles are thicker than the other.

The following morphometric characters were measured, indicating in parenthesis the measurement as defined by Roonwal (1970): LH, length of head (9), LN, length of nasus (13), WH, width of head (18), LLM, length of left mandible (36), WP, maximum width of pronotum (68), LT, length of hind tibia (85). The distribution map was created using Quantum GIS 2.8.3.

## Taxonomy

### Uncitermes almeriae

Taxon classificationAnimaliaIsopteraTermitidae

Carrijo sp. n.

http://zoobank.org/093682C6-EDDD-4E9E-9155-43439AB1AB0C

#### Type-locality.

ECUADOR. Orellana: Francisco de Orellana, Yasuni National Park, -0.6717, -76.3979.

#### Holotype.

Soldier. 31.v.2011, R.H.Scheffrahn col., MZUSP 23117.

#### Paratypes.

ECUADOR. **MZUSP 23117**, same sample of holotype (2 soldiers, 13 workers); **UF EC1066**, same sample of holotype (16 soldiers, 20 workers); **UF EC1000**, same data of holotype (8 soldiers, 16 workers); PERU. **UF PU311**, 21 km South of Ciudad Constitución,-10.04915, -75.02859, 27.v.2014, R.H.Scheffrahn col. (17 soldiers, 12 workers); **MZUSP 23236**, duplicate of the previous (2 soldiers and 3 workers).

*Imago*. Unknown.

**Figure 1.**
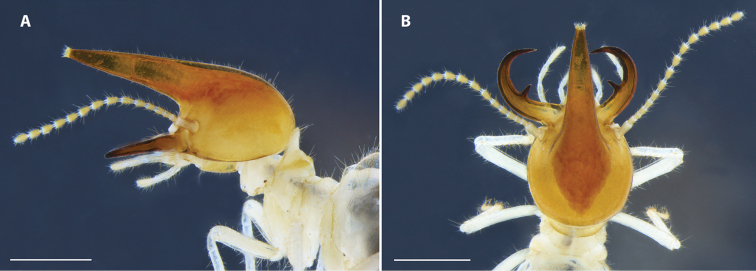
Soldier head capsule of *Uncitermes almeriae* sp. n. **A** lateral view **B** dorsal view. Scale: 1 mm.

*Soldier* (Fig. 1). Monomorphic. Head capsule, in dorsal view, rounded. Antennae with 15 articles; relative length formula 1>2>3=4<5. In lateral view, nasus narrowly conical, forming a ca. 30° angle with base of head; dorsal margin of nasus undemarcated with margin of vertex and evenly curving along with occipital margin. Labrum rounded, anterior margin hyaline, without distinct tip. Postmentum subrectangular. Mandibles symmetrical, piercing, and hooked; narrow tooth subconical and approximately central on the inner margin. Molar region absent. When closed, in dorsal view, mandibles overlap nearly completely. Pronotum with length of anterior lobe longer than posterior lobe. Tibial spur formula 2:2:2. Head capsule covered of short bristles and sparse long bristles. Nasus covered of many short bristles and tip of nasus crowned with short bristles. Pronotum with long and short bristles along margins, denser on lateral margins, some short bristles on anterior lobe. Meso- and metanotum with long and short bristles on posterior margins; tergites and sternites completely covered by a dense layer of long bristles. Legs with many long and short bristles; thick bristles on inner face of tibia. Range and mean of measurements (mm) of six soldiers from three colonies: LH 1.30–1.40 (1.35); LN 1.43–1.58 (1.50); LLM 0.93–1.00 (0.96); WH 1.35–1.43 (1.38); WP 0.80–0.88(0.84); LT 1.68–1.80 (1.74).

#### Remarks.

**Figure 2.**
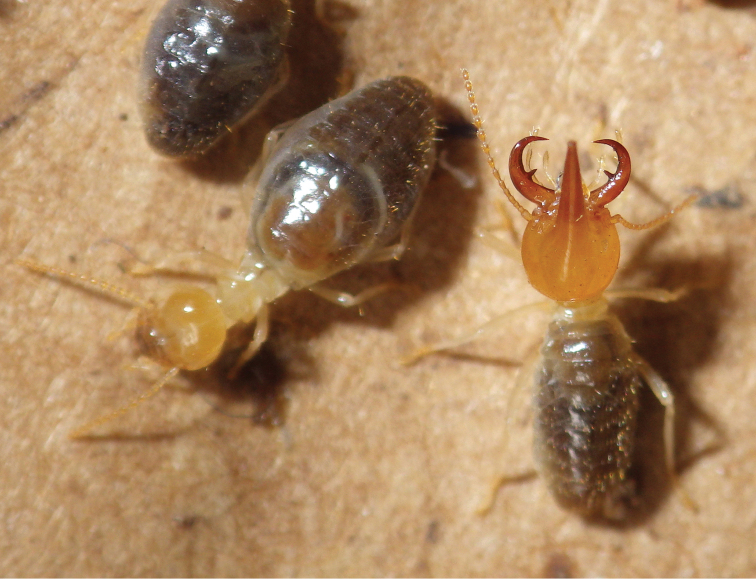
Live habitus of *Uncitermes almeriae* sp. n.

When alive (Fig. 2) the frontal gland coloration of both *Uncitermes* spp. is the same as the surrounding head capsule. After ethanol preservation, the frontal gland of both species turns to a dark reddish color in contrast to the remainder of the head capsule. In some preserved soldiers, a clear, hardened defensive secretion can be seen as droplets clinging to the nasus. Unlike some Nasutitermitinae that squirt their secretion several body lengths, this genus and probably all syntermitids exude their defensive secretion with little force.

**Figure 3.**
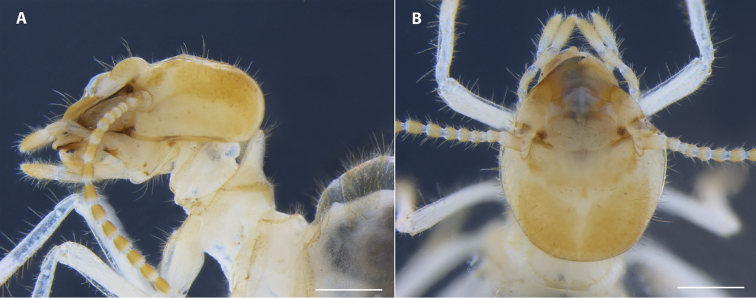
Worker head capsule of *Uncitermes almeriae* sp. n. **A** lateral view **B** dorsal view. Scale: 0.5 mm.

**Figure 4.**
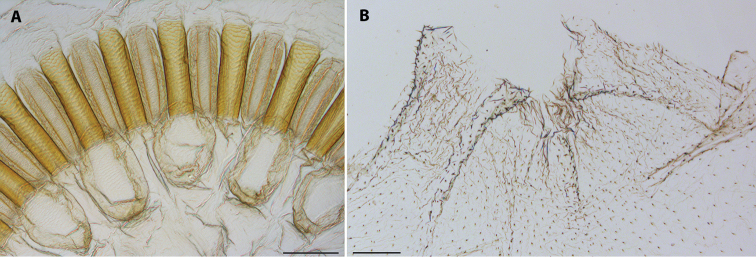
Digestive tube of worker of *Uncitermes almeriae* sp. n. **A** gizzard **B** enteric valve. Scale: 0.1 mm.

*Worker* (Figs 3, 4). As described and illustrated for genus by Rocha et al. (2012). Range and mean of measurements (mm) of twelve workers from three colonies: LH 0.75 – 1.00 (0.94); WH 1.13 – 1.30 (1.21); LT 1.33 – 1.50 (1.43).

#### Etymology.

The species name is a latinized noun in the genitive case. *Uncitermes almeriae* sp. n. is named in honour of Almeri Fernandes Sousa, TFC’s mother.

#### Biological notes.

There are no biological data published for *Uncitermes almeriae* sp. n. However, *Uncitermes teevani* is commonly sampled in rotten wood, litter, soil, dry palm tree stipes and clumps of roots, probably foraging in a soil-litter interface. There is a report of a nest of *Uncitermes teevani* from French Guiana under a dead trunk (in the hollow cylinder section), the nest structure was about a meter long, and the royal cell was attached to the wood, asymmetrically in the oval shaped nest structure (Jan Šobotník, personal communication).

#### Comparisons.

The soldier of *Uncitermes almeriae* sp. n. has short bristles covering all the head capsule and frontal tube, while *Uncitermes teevani* has only sparse long bristles on head capsule and the frontal tube is glabrous.

#### New records of *Uncitermes teevani*

**Figure 5.**
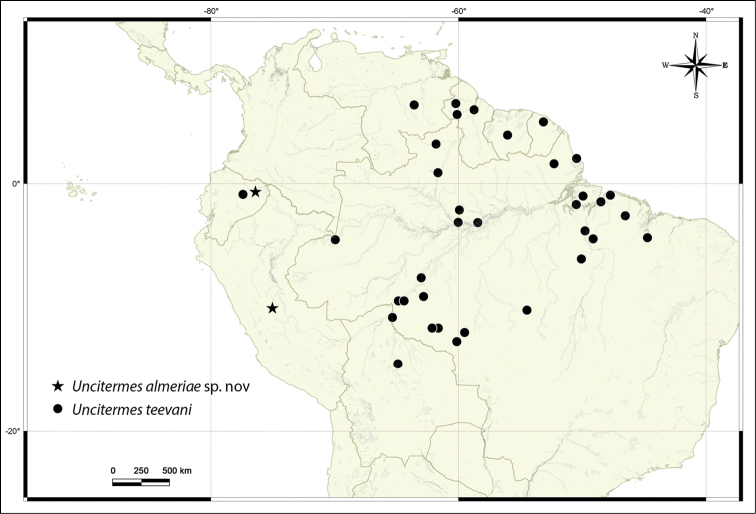
Distribution map of *Uncitermes* spp. Data for *Uncitermes teevani* available in Davies (2003), Rocha et al. (2012), Carrijo (2013) and this paper.

(Fig. 5). BOLIVIA. **UF BO458**, N. San Javier, -14.5491, -64.8896, 29.v.2013, J.A. Chase col. ECUADOR. **UF EC210**, Yasuni National Park (P.U.C.E. Research Station), 20.iii.2006, B.W. Bahder col. **UF EC999**, 1001, 31.v.2011, J.R. Mangold col. **UF EC1163, 1176, 1177**, Tiputini river, stop 2, -0.67530, -76.36864, 01.vi.2011.

## Supplementary Material

XML Treatment for Uncitermes almeriae
